# The Role of (*E*)-2-octenyl Acetate as a Pheromone of *Bagrada hilaris* (Burmeister): Laboratory and Field Evaluation

**DOI:** 10.3390/insects11020109

**Published:** 2020-02-09

**Authors:** Mokhtar Abdulsattar Arif, Salvatore Guarino, Stefano Colazza, Ezio Peri

**Affiliations:** 1 Dipartimento di Scienze Agrarie, Alimentari e Forestali, Università degli Studi di Palermo, Viale delle Science Ed. 5, 90128 Palermo, Italy; mokhtar.arif@unipa.it (M.A.A.); stefano.colazza@unipa.it (S.C.); ezio.peri@unipa.it (E.P.); 2 Institute of Biosciences and Bioresources (IBBR), National Research Council of Italy (CNR), Corso Calatafimi 414, 90129 Palermo, Italy

**Keywords:** painted bug, olfactometer, EAG, traps

## Abstract

The pentatomid bug *Bagrada hilaris* is a key pest of brassicaceous crops in several areas of the world. Previous studies suggest that mate location of this species is mediated by volatile chemicals produced by males, among which the main compound is (*E*)-2-octenyl acetate. However, the possible attraction of males, females, and nymphs to this compound has not yet been specifically tested. In this study, we tested the response of *B. hilaris* females, males, and nymphs to (*E*)-2-octenyl acetate using an electroantennogram (EAG) and olfactometer in the presence or absence of a host plant. Moreover, (*E*)-2-octenyl acetate as an attractant lure in field trap bioassays was evaluated. EAG recordings showed that this compound evokes antennal responses in *B. hilaris* females. Olfactometer behavioral responses showed that females and nymphs were attracted to (*E*)-2-octenyl acetate, while males showed no attraction. In the field trap bioassays, captures were obtained in traps baited with 5 and 10 mg of (*E*)-2-octenyl acetate, while in traps loaded with 2 mg and control traps, there were no recorded catches. These results suggest the involvement of (*E*)-2-octenyl acetate in intraspecific interactions of this species.

## 1. Introduction

The painted bug, *Bagrada hilaris* (Burmeister), is an invasive stink bug which feeds mainly on brassicaceous hosts, and it is particularly detrimental to crops in recently invaded areas [1]. This species is widely distributed across Africa, southern and central Europe, Pakistan, India, China, and parts of Southeast Asia [2,3]. In 2008, the painted bug was reported in California, probably introduced by commercial trade, and it rapidly expanded its range to the brassicaceous crops of coastal California and central Arizona [4] and then to Nevada, New Mexico, and Utah [5]. More recently, *B. hilaris* has been reported in Mexico [6], Hawaii [7], and Chile [8]. *Bagrada hilaris* has had a remarkable impact on agriculture in the Americas; it has been estimated that about 90% of the cole crops including broccoli, cabbage, and cauliflower acreage planted in the USA have been infested by the painted bug, with yield losses often exceeding 10% of production [1]. Painted bug infestation has severe physiological consequences on host plants; for example, it reduces leaf photosynthesis of cauliflower plants by about 40% [9]. The control of this pest is difficult, and to date, there are no effective detection strategies or monitoring tools for the pest [3,10]. 

Several studies have focused on chemical communication in stink bugs, including the identification of sex pheromones [11,12,13,14,15,16]. The great majority of pheromones described for these species are produced by adult males [17,18], although nymphs are known to produce aggregation compounds [19].

In the case of *B. hilaris*, (*E*)-2-octenyl acetate, the main compound produced by adults, with significantly higher amounts emitted by males in comparison with females, was reported as a putative sex pheromone, as it might be involved in the attraction process of females to males [20]. Although Bundy et al. [3] reported that attempts to use this compound as a monitoring tool were not successful, the attraction response of *B. hilaris* individuals to (*E*)-2-octenyl acetate has yet to be tested. 

In this study, the electroantennographic and behavioral responses of *B. hilaris* to (*E*)-2-octenyl acetate were assessed in order to establish the suitability of this semiochemical for further trials. In laboratory experiments, we evaluated the antennal response of *B. hilaris* females, males, and nymphs to (*E*)-2-octenyl acetate using electrophysiological techniques. Furthermore, the attraction response elicited from (*E*)-2-octenyl acetate alone or in the presence of a host plant using a two-choice olfactometer was evaluated. Finally, in experiments carried out in the field, we tested (*E*)-2-octenyl acetate as a lure in traps that were specifically designed.

## 2. Materials and Methods 

### 2.1. Insects and Plants

A colony of *B. hilaris* was established and restocked regularly with individuals collected from caper (*Capparis spinosa* L.) fields on the island of Pantelleria (Trapani, Italy). Insects were reared in an environmentally controlled room (30 ± 2 °C, 70 ± 10% relative humidity (RH), photoperiod 16L:8D) in wooden cages (25 × 25 × 40 cm) with two 5-cm diameter mesh-covered holes for ventilation. The colony was fed with cauliflower and cabbage plants, depending on seasonal availability. As *B. hilaris* females oviposit in the soil, dishes (6-cm Ø) with a mixture of sand, silt, and clay (33% of each soil component) were placed in the cages as oviposition sites. Dishes were changed weekly, and those with eggs were kept in separate cages until the emergence of nymphs. Adults and nymphs (4th and 5th stages) were used separately in experiments.

Seeds of *Brassica oleracea* var. botrytis L. (cauliflower) obtained from a local market (Palermo, Italy) were placed on cotton wool (5 g) soaked with distilled water and held in glass containers with a distance of circa 0.5 cm between seeds to obtain a seedling cluster of 50 individuals. The containers were placed in an environmentally controlled growth chamber (25 ± 1 °C, 70 ± 10% RH, photoperiod 16L:8D). After 7 days, newly emerged seedling clusters were used in behavioral experiments. The choice of this host plant species at the seedling stage as an attractant source for behavioral experiments was suggested by a recent study carried out by Guarino et al. [20].

### 2.2. Electroantennography (EAG)

Dose-response experiments using (*E*)-2-octenyl acetate were carried out. Electroantennograms (EAG) were conducted using aliquots of 2 μL of test solution of (*E*)-2-octenyl acetate (Bedoukian Research, Danbury, CT, USA) dissolved in acetone, pipetted onto a piece of filter paper (Whatman, grade 1). Tested doses of (*E*)-2-octenyl acetate were 0.02, 0.2, 2, 20, and 200 µg; acetone was used as a control. The loaded paper was exposed to the air for 40 s to allow the solvent to evaporate and was then placed inside a glass Pasteur pipette. Puff stimuli were blown into an airstream that passed over the antennal preparation using a flow controller (model CS-05; Syntech, Hilversum, the Netherlands) to generate a 1.5-s stimulus at 1-min intervals, with a flow rate of 1.5 L/min. The signals generated by the antennae were passed through a high-impedance amplifier (model IDAC-4, Syntech) and were recorded with custom software (Syntech, Hilversum, the Netherlands). For the EAG preparations, adult and nymphs were anesthetized by refrigeration at −4°C for 40 s; the head was excised and mounted on a glass capillary tube reference electrode (1.5 mm diameter) filled with 0.1 M KCl solution and connected with silver wire to the amplifier. The recording electrode was a similar glass capillary in contact with the tip of the antenna. The capillary tubes were drawn to a fine point using a microelectrode puller (PC-10, Narishige, Tokio, Japan) to achieve a diameter enabling insertion into the antennal tip.

### 2.3. Open Vertical Y-Shaped Olfactometer

Bioassays were conducted with an open vertical Y-shaped olfactometer consisting of a brass rod (left and right arms 20 cm long, central arm 25 cm long, 1 cm diameter).

The behavioral bioassays were conducted using (*E*)-2-octenyl acetate alone or in the presence of the host plant in two separate experiments. In the first experiment, (*E*)-2-octenyl acetate was tested alone versus air used as a control. In the second experiment, (*E*)-2-octenyl acetate was assessed in the presence of a 7-day-old seedling cluster of *B. oleracea* var botrytis versus a seedling cluster alone used as the control. (*E*)-2-octenyl acetate was applied using 2 µL of a 10% acetone solution (200 µg) on a filter paper disk (Whatman N. 1) and left for 5 min for evaporation of the solvent before being placed in the test jar. The test stimuli were changed after 15 min. The left and right arms were covered with two glass tubes (18 cm long, 5 cm diameter) terminating in hose nipples connected by Tygon tubes to a high-purity air source, and air flow was controlled with a flow meter at a rate of 0.2 L/min. The air flowed through two glass chambers (125 mL each), which held the test stimuli. Light was provided with a halogen lamp (12V–35W, Osram, Münich, Germany) hanging 30 cm above the olfactometer. Experiments were carried out under ambient laboratory temperature and humidity conditions (25 ± 1 °C, and 50 ± 15% RH). For each replicate, a single adult or nymph was gently placed at the bottom of the central arm of the olfactometer with a paint brush and allowed 10 min to respond. The bugs moved from the bottom upward toward the light source and upon arriving at the Y junction, chose between the two different volatile stimuli. The criterion for a response was that the test bug walked in the test arm or the control arm for at least 5 cm past the Y junction (first choice). Bugs that did not move into one of the two arms during the 10 min trial were scored as non-responders and were not included in the analysis. After 8 replicates, the glass parts of the apparatus were washed with water and detergent and then wiped with acetone, and the brass rod was cleaned with distilled water and acetone and baked at 200 °C for 60 min. Experiments were carried out from 2:00 p.m. to 7:00 p.m.

### 2.4. Field Bioassay

Field bioassays using (*E*)-2-octenyl acetate were conducted in a caper orchard on Pantelleria Island (Italy) (36°46′15.2″ N 11°57′44.1″ E), infested with *B. hilaris*. *Bagrada hilaris* aggregates on the host plant and oviposits in the soil; this provoked the use of a novel two-entrance trap, designed to be placed on the ground and in the proximity of the host plant. We used a new type of horizontal, dual funnel-sided plastic trap (25 × 15 × 15 cm) furnished by GEA S.r.L., (Settimo Milanese Milan, Italy) (see Figure S1). Paraffin oil was applied on the board of the inner part of the funnels to prevent insect escape. Traps were placed in the proximity of the caper plants and were partially buried in order to facilitate insect entrance and to prevent wind damage. Each trap was baited with a 0.5 mL polyethylene tube containing 2, 5, or 10 mg of (*E*)-2-octenyl acetate dissolved in 100 µL of acetone. The emission rate estimation of (*E*)-2-octenyl acetate was previously carried out in the laboratory in order to assess the amount of the compound emitted with time. The polyethylene tubes were loaded with the same dose tested in the field (n = 3 per each dose). The (*E*)-2-octenyl acetate emitted from polyethylene tubes was collected by placing the dispensers in an air entrainment cylindrical glass chamber (25 mL volume) where charcoal-filtered air passed through at 300 mL/min. A glass tube containing a plug of 100 mg of Porapak Q (80–100 mesh; Sigma–Aldrich, Milan, Italy) was used to collect the (*E*)-2-octenyl acetate emitted on different days from the polyethylene dispenser loading. After collection for 15 min, the traps were eluted with 1 mL of acetone. The collections were carried out after one day from the dispenser loading and thereafter every three days for 3 weeks. All replicates were carried out in a temperature-controlled room (25 ± 1°C). Extracts were stored at 4 °C in glass vials with Teflon cap liners until used for GC/MS analyses. GC-MS analyses were performed on an Agilent 6890 GC system interfaced with an MS5973 quadruple mass spectrometer. One μL of extract was injected onto a DB5-MS column in splitless mode. Injector and detector temperatures were 260 °C and 280 °C, respectively. Helium was used as the carrier gas. The GC oven temperature was set at 40 °C for 5 min and then increased by 10 °C/min to 250 °C. Electron impact ionization spectra were obtained at 70 eV, with recording mass spectra from 40 to 550 amu. In order to estimate the quantitative amounts of (*E*)-2-octenyl acetate emitted from the polyethylene tube, the integrated GC peaks were compared with a calibration curve generated with standard solutions. Linearity was determined for this compound by injecting GC concentrations of 6, 12, 25, 50, and 100 ng μL^−1^, generating a calibration curve that had regression coefficients (*R*^2^) of 0.997.

The distance between traps was approximately 8 m. Three traps per dose and three acetone-only bait control traps were placed in the field using a Latin square design. Traps were inspected every 3 days for 15 days, and the number of trapped individuals (males, females, or nymphs) was recorded during each inspection.

### 2.5. Statistics and Data Analyses

EAG data, presented as the means of antennal responses in mV after subtraction of the responses to the solvent were root square transformed and analyzed using one-way ANOVA. Fisher’s least significant difference analysis (LSD) was used to distinguish differences among means for each dose and gender/life stage. Vertical Y-shaped olfactometer choice experiments were analyzed using *χ*^2^ tests. Data of the emission curve of (*E*)-octenyl acetate from polyethylene dispensers loaded with different doses were compared for each day of sampling by one-way ANOVA, followed by Tukey’s test. For field experiments, the numbers of captured adults and nymphs were compared using a nonparametric Kruskal–Wallis analysis of variance. All statistical analyses were performed using Statistica 10.0 for Windows (Statsoft 2001, Vigonza, PD, Italy). 

## 3. Results

### 3.1. Electroantennography (EAG)

**Figure 1:**
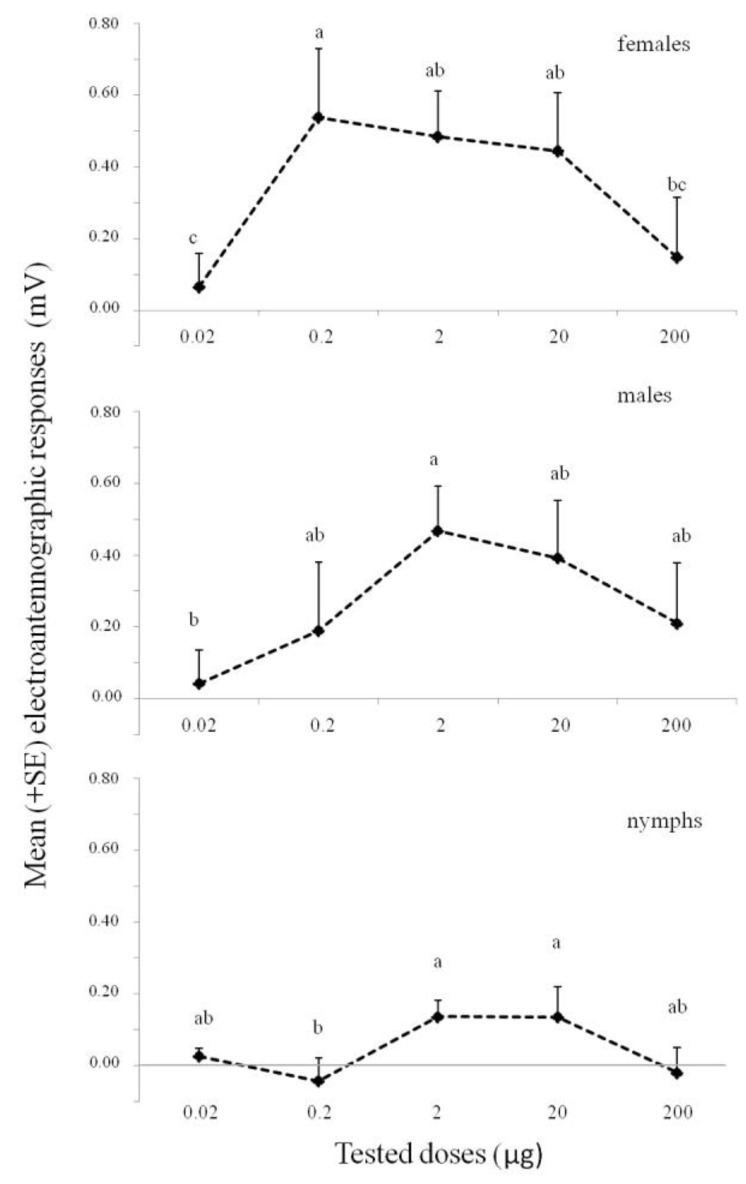
EAG dose–response curves of *Bagrada hilaris* individuals (female n = 7; male n = 7; nymphs n = 6) to (*E*)-2-octenyl acetate at different doses. EAG amplitudes were adjusted to a control stimulus (acetone). Different letters indicate that values differ statistically at *p* < 0.05 (ANOVA, followed by LSD test).

The results of the EAG experiments are shown in Figure 1. Overall, the results obtained show that antennae of females, males, and nymphs exhibited dose-dependent responses to (*E*)-2-octenyl acetate but with a different sensitivity. In particular, females’ antennae exhibited a significant electroantennographic response at 0.2 µg (*p* < 0.05; ANOVA followed by LSD test), and similar responses were observed at 2 and 20 µg. In males, the only significant response occurred at 2 µg (*p* < 0.05; ANOVA followed by LSD test); however, it was not significantly different from 0.2, 20, and 200 µg doses. Nymphs showed a significant response at the doses of 2 µg and 20 µg (*p* < 0.05; ANOVA followed by LSD test). Antennae of males, females, and nymphs reached saturation with increasing doses and exhibited a decreasing response at the highest dose used (200 µg). 

### 3.2. Open Vertical Y-Shaped Olfactometer

**Figure 2:**
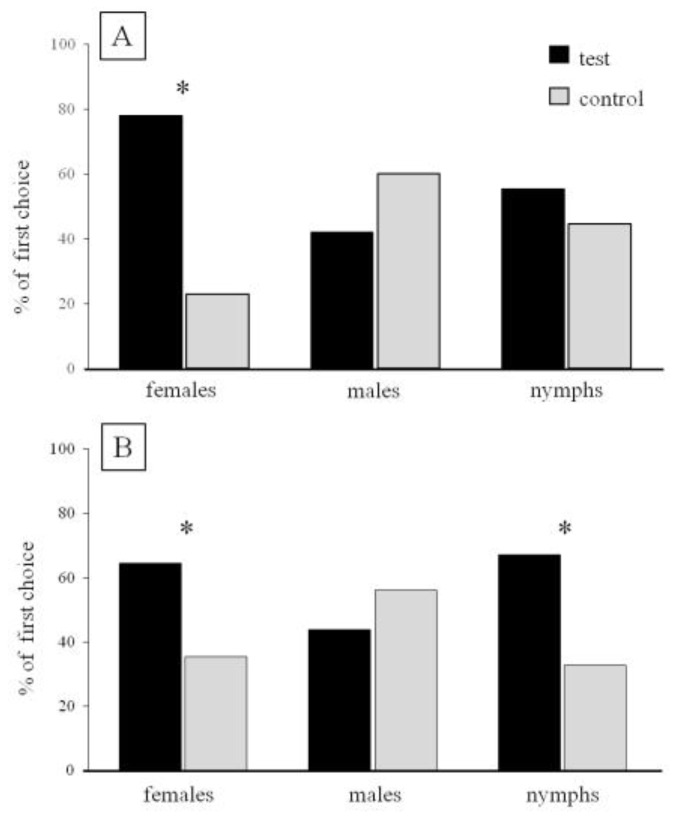
**(A)** Response of *Bagrada hilaris* individuals to (*E*)-2-octenyl acetate (test) vs. the solvent (control) (females n = 46; males n = 36; nymphs n = 56); (**B)** Response of *B. hilaris* individuals to (*E*)-2-octenyl acetate in the presence of *Brassica oleracea* seedlings (test) versus *B. oleracea* seedlings alone (control) (females n = 62; males n = 66; nymphs n = 61); * *p* < 0.05.

Responses of *B. hilaris* adults and nymphs in open vertical Y-shaped olfactometer bioassays are shown in Figure 2. In the first experiment, females were attracted by (*E*)-2-octenyl acetate rather than air (*χ*^2^ = 10.52, df = 1, *p* < 0.001, n = 46) while males and nymphs were not attracted (*χ*^2^ = 0.71, df = 1, *p* = NS, n = 36; *χ*^2^ = 0.64, df = 1, *p* = NS, n = 56, respectively) (Figure 2A). In the second experiment, conducted in the presence of *B. oleracea* seedlings in both test and control arms, there was a significant attraction response to the (*E*)-2-octenyl acetate rather to seedlings alone by both female (*χ*^2^ = 5.22, df = 1, *p* = 0.02, n = 62) and nymphs (*χ*^2^ = 7.22, df = 1, *p* = 0.07, n = 61). Males were not attracted to (*E*)-2-octenyl acetate (χ^2^ = 0.96, df = 1, *p* = 0.3, n = 66) (Figure 2B).

### 3.3. Field Bioassay

**Figure 3:**
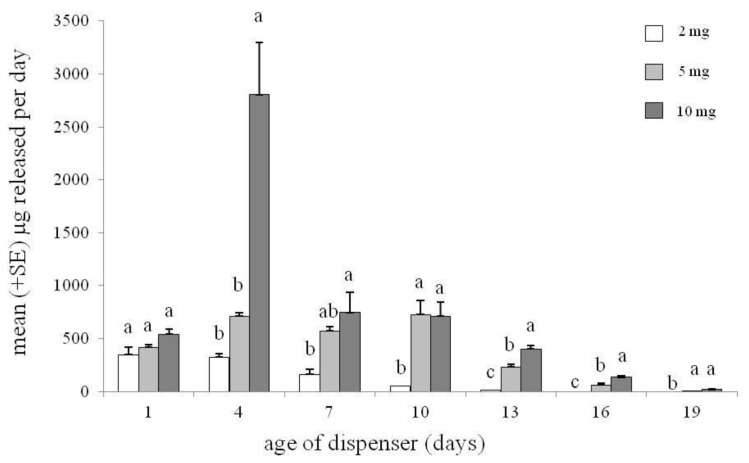
Emission rate of (*E*)-2-octenyl acetate from polyethylene tube dispensers loaded with doses of 2, 5, and 10 mg. Different letters within the same day of sampling indicate statistical differences between doses at *p* < 0.01 (ANOVA, followed by Tukey’s test).

The (*E*)-2-octenyl acetate emission rate (ng/hr) from the polyethylene dispensers is reported in Figure 3. One day after the dispenser preparation, the emission of (*E*)-2-octenyl acetate was similar among the different treatments. However, the dispensers loaded with different doses of (*E*)-2-octenyl acetate emitted the substance with a different pattern during the experiment. In detail, on the fourth day after releaser loading, the dispensers with 10 mg emitted an amount of (*E*)-2-octenyl acetate that was 8- and 4-fold higher than that of 2 and 5 mg, respectively (*p* < 0.01; Tukey test). On days seven and ten, the dispensers loaded with 10 mg emitted a higher amount of (*E*)-2-octenyl acetate than the dispensers loaded with 2 mg (*p* < 0.01; Tukey test) but similar to the ones loaded with 5 mg (*p* = NS). On days thirteen and sixteen, the amount emitted was higher from the 10 mg dispenser compared with the others (*p* < 0.01; Tukey test). However, the amount emitted from the 5 mg dispenser was higher than that emitted from the 2 mg dispenser (*p* < 0.01; Tukey test). Finally, on day nineteen, the 10 and 5 mg dispensers emitted a similar amount of (*E*)-2-octenyl acetate that was higher than that emitted from the 2 mg dispensers (*p* < 0.01; Tukey test).

**Figure 4:**
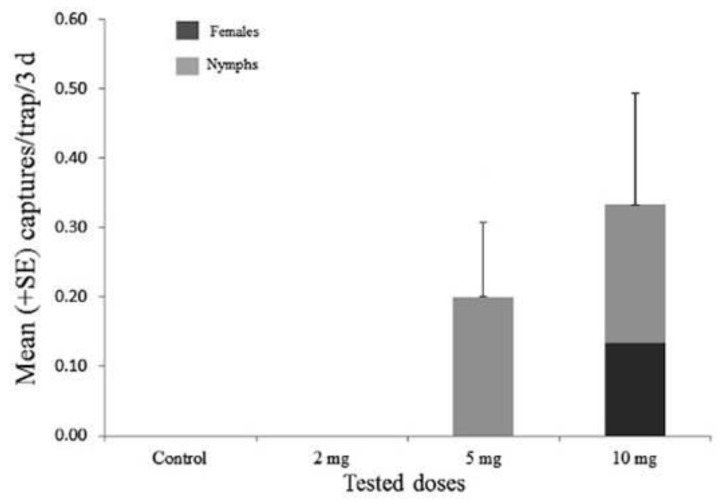
Mean number (+SE) of *Bagrada hilaris* individuals captured by traps baited with (*E*)-2-octenyl acetate at different doses and the solvent (control); *χ*^2^= 8.24; df = 3; *p* < 0.05.

The results of the field bioassays with (*E*)-2-octenyl acetate are reported in Figure 4. The number of *B. hilaris* individuals captured during the field experiment was significantly different among treatments, i.e., doses of (*E*)-2-octenyl acetate (*χ*^2^= 8.24; df = 3; *p* < 0.05). The traps loaded with 10 mg captured the highest number of individuals overall, with a mean of 0.33 ± 0.15 individuals per trap every three days, catching both females and nymphs. In the traps baited with 5 mg of (*E*)-2-octenyl acetate, only *B. hilaris* nymphs were caught (mean 0.2 ± 0.1). No captures were observed in the traps baited with 2 mg of (*E*)-2-octenyl acetate and in the control traps. 

## 4. Discussion

The results of the experiments conducted in this study suggest that (*E*)-2-octenyl acetate is a *B. hilaris* pheromone, playing the role of a sex pheromone of males and females and an aggregation pheromone of nymphs.

The data obtained from EAG experiments indicated a higher sensitivity of *B. hilaris* female antenna to (*E*)-2-octenyl acetate in comparison with males and nymphs. A similar strong sensitivity of female antenna has been reported in other stink bug species, i.e., in EAG experiments testing the sex pheromone in *Nezara viridula* (L.) [21], *Thyanta pallidovirens* (Stål) [22], *Chinavia ubica* (Rolston), and *C. impicticornis* (Stål) [23]. 

The results of EAG bioassays were confirmed by the bioassays carried out in a vertical Y-shaped olfactometer that indicated the attraction response of females toward (*E*)-2-octenyl acetate but no response from males. In the same experiment, nymphs were attracted to (*E*)-2-octenyl acetate only in presence of the host plant, suggesting that host plant volatiles can augment the response to the pheromone, as observed in other stink bugs such as *Euschistus conspersus* Uhler [24] and *Halyomorpha halys* Stål [25].

The field experiments confirmed the attractant role of this compound with the presence of captures in the traps baited with two highest doses of (*E*)-2-octenyl acetate tested. The overall low number of individuals captured in the traps in our experiments might be influenced by the complex behavior of stink bugs that has often led to disappointing results in field tests in other cases. In fact, similar difficulties have been observed during trials to bioassay possible pheromone components for other phytophagous bug species [26]. For example, although the *N. viridula* pheromone had strong attraction towards females in laboratory bioassays [11,27], field tests results have often not been confirmatory [13,28]. In several cases, the stink bug pheromone attracts the insect in the proximity of the traps, but the number of the individuals entering is poor [25,29]. Moreover, several papers suggest that the commonly used insect trap designs are not effective for phytophagous stink bugs due to inadequate trap design [30,31]. Moreover, the release rate from the polyethylene dispensers loaded with different doses of (*E*)-2-octenyl acetate showed a rapid exhaustion from the 2 mg dispenser in comparison with the 5 and 10 mg dispensers. Consequently, the different emission rate from the releasers loaded with different doses of pheromone could have determined the different number of captures recorded in the field, especially if the attraction response of *B. hilaris* females and nymphs to (*E*)-2-octenyl acetate is linked with the amount perceived.

Overall, our laboratory and field bioassays support the previous study of Guarino et al. [20], who first suggested a possible pheromonal role of (*E*)-2-octenyl acetate for *B. hilaris*. In detail, seems that, similar to all the other pentatomid bugs, *B. hilaris* female adults respond to a semiochemical that results in close proximity of the two sexes. Once sexes are in close proximity, other close-range courtship behavior can take place, including contact cues, as observed by Guarino et al. [20]. However, unlike other pentatomid bugs, the attraction is not determined by a specific compound emitted by mature males, but by a compound produced by both sexes [20] as well as the nymphs (Guarino, personal observation). Moreover, in other pentatomid bugs, male-produced compounds act as sex pheromones attracting only females, such as in *Piezodorus guildinii* (Westwood) [32], *T. pallidovirens* [33], *Oebalus poecilus* (Dallas) [34], *Eysarcoris parvus* (Uhler) [35], and *Pallantia macunaima* (Grazia) [36]. In other cases, the male-produced compounds act as aggregation pheromones, attracting females, males, and even nymphs, such as in *Murgantia histrionica* (Hahn) [37,38], *N. viridula* [27], *Eysarcoris lewisi* (Distant) [39], *Euschistus tristigmus* (Say) [18,30], and *H. halys* [40].

However, in the case of *B. hilaris*, the attraction behavior within conspecifics seems to be driven by a pheromone produced by both sexes and at all life stages (Guarino, personal observation), albeit in different amounts but eliciting attraction only of females and nymphs. This, together with the peculiar oviposition behavior in the soil, makes the behavior of *B. hilaris* rather unique among pentatomids.

The fact that (*E*)-2-octenyl acetate is produced through the entire lifetime of *B. hilaris* is not surprising as this presence of a qualitative similar compound emission in adults and nymphs has been observed in other stink bugs [41,42]. Moreover, the lack of distinct differences in compounds produced by the two sexes has been already observed in other Heteroptera species such as *Lygus lineolaris* (Palisot de Beauvois) [12]. (*E*)-2-octenyl acetate is present in other Heteroptera species, often with a defensive role, but in a few cases as a sex pheromone [12,18]. For example, in the lygaeid *Geocoris punctipes* (Say), (*E*)-2-octenyl acetate is produced mainly by females and it plays a role as a sex pheromone [43]. Furthermore, in the alydid *Leptocorisa chinensis* (Dallas)*,* a blend of (*E*)-2-octenyl acetate and octenol is produced by both sexes, but it is only attractive to males [44]. Moreover, (*E*)-2-octenyl acetate, together with other compounds, was found in the metathoracic gland contents and in aeration extracts of other stink bugs such as *T. pallidovirens* and *Euschistus heros* (F.) [33,45]. In fact, in several cases, (*E*)-2-octenyl acetate is considered part of the defensive blend of Heteroptera species, having a repellent effect toward predators [46]. Defensive compounds have been found to function as alarm pheromones [47] or even more specifically as sex pheromones [48,49,50]. The use of a chemical substance for two or more functions is well-known and has been referred to as “semiochemical parsimony” [51].

## 5. Conclusions

To conclude, the results obtained suggest that (*E*)-2-octenyl acetate is involved in intraspecific communication of *B. hilaris*, attracting females and nymphs. The weak attraction observed in the field tests suggests that this semiochemical is probably not the only cue involved in the intra-specific communication of this species. In several phytophagous stink bugs, mating and aggregation behaviors are also influenced by other sensory cues that act at short range, such as visual and/or vibrational signals [52,53].

Our future efforts to develop an effective lure for this species will focus on testing (*E*)-2-octenyl acetate together with plant-derived attractant compounds recently identified in the host plant such as the novel identified diterpene hydrocarbon found in seedling stages of *Brassica* spp. [10].

## Supplementary Materials

The following are available online at https://www.mdpi.com/2075-4450/11/2/109/s1, Figure S1: Horizontal, dual funnel-sided plastic traps (25 × 15 × 15 cm) furnished by GEA S.r.L., (Settimo Milanese Milan, Italy) and used for the field experiments.
