# Supplementary figures and images for: The Role of (E)-2-octenyl Acetate as a Pheromone of Bagrada hilaris (Burmeister): Laboratory and Field Evaluation
Source: Insects. 2020 Feb 9;11(2):109. doi: 10.3390/insects11020109 (PMC7074293; doi:10.3390/insects11020109)

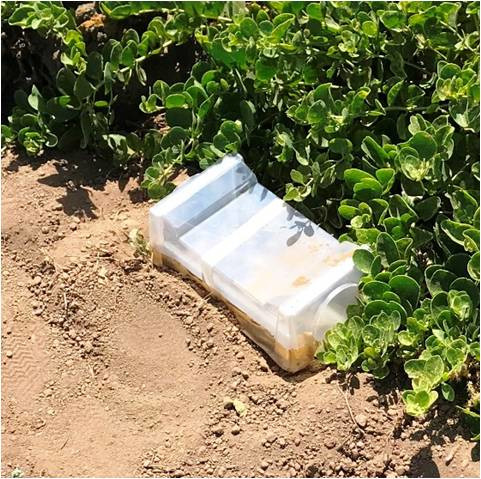

Supplement: Supplementary file 1 [file insects-11-00109-s001.zip › insects-706726-supplementary.jpg]
